# Meningioma Genomics: Diagnostic, Prognostic, and Therapeutic Applications

**DOI:** 10.3389/fsurg.2016.00040

**Published:** 2016-07-06

**Authors:** Wenya Linda Bi, Michael Zhang, Winona W. Wu, Yu Mei, Ian F. Dunn

**Affiliations:** ^1^Department of Neurosurgery, Brigham and Women’s Hospital, Harvard Medical School, Boston, MA, USA

**Keywords:** meningioma, genomics, molecular taxonomy, targeted therapy, precision medicine

## Abstract

There has been a recent revolution in our understanding of the genetic factors that drive meningioma, punctuating an equilibrium that has existed since Cushing’s germinal studies nearly a century ago. A growing appreciation that meningiomas share similar biologic features with other malignancies has allowed extrapolation of management strategies and lessons from intra-axial central nervous system neoplasms and systemic cancers to meningiomas. These features include a natural proclivity for invasion, frequent intratumoral heterogeneity, and correlation between biologic profile and clinical behavior. Next-generation sequencing has characterized recurrent somatic mutations in *NF2, TRAF7, KLF4, AKT1, SMO*, and *PIK3CA*, which are collectively present in ~80% of sporadic meningiomas. Genomic features of meningioma further associate with tumor location, histologic subtype, and possibly clinical behavior. Such genomic decryption, along with advances in targeted pharmacotherapy, provides a maturing integrated view of meningiomas. We review recent advances in meningioma genomics and probe their potential applications in diagnostic, therapeutic, and prognostic frontiers.

Meningioma genetics are undergoing a revolution in taxonomy and molecular stratification, punctuating an equilibrium that has existed since Cushing’s germinal studies nearly a century ago (1). The understanding of meningiomas rests on a growing appreciation that these tumors share similar features with other intra-axial central nervous system (CNS) neoplasms as well as systemic cancers. Moreover, maturing technologies in genomics and immunotherapy are increasingly intersecting to provide an integrated view on meningioma biology. We review recent advances in meningioma genomics and probe their potential applications in diagnostic, therapeutic, and prognostic frontiers.

## Meningioma Histopathologic Classification

Meningiomas account for over a third of all primary CNS tumors diagnosed in the United States, with ~18,000 new cases diagnosed annually and a prevalence of 97.5/100,000 individuals, making them the most common primary intracranial neoplasms in adults (2, 3). Most meningiomas are considered benign. A small, but growing, proportion display aggressive behavior characterized by invasive growth patterns and higher rates of recurrence (4).

**Figure 1:**
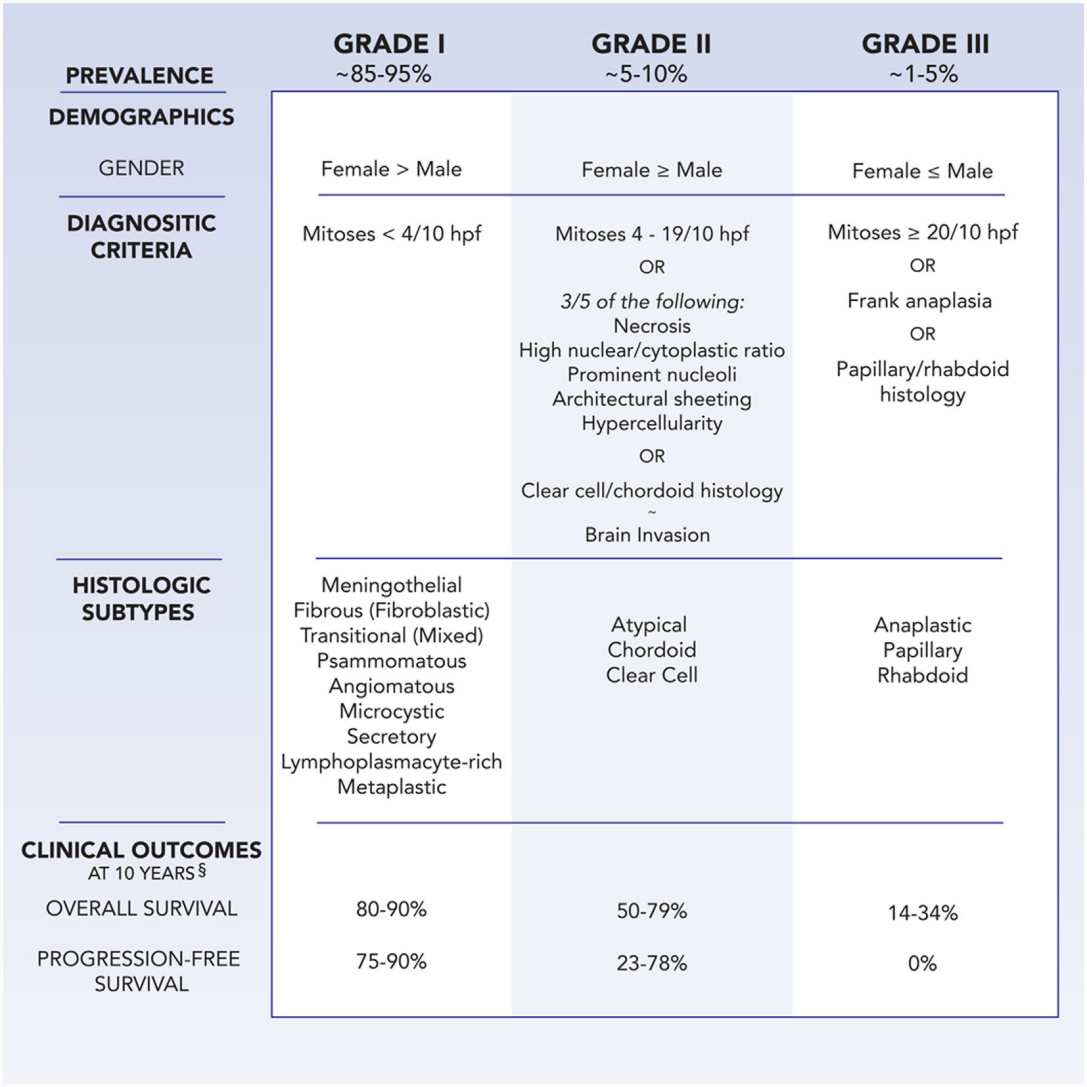
**Demographics, WHO diagnostic criteria, histologic subtypes, and clinical outcomes at 10 years follow-up for meningioma, as stratified by grade**. ^§^Clinical outcomes are influenced by age, comorbidities, extent of resection, adjuvant therapy, and tumor location.

Meningiomas are classified by the World Health Organization (WHO) system as grades I, II, and III, with higher grades associated with greater rates of morbidity and mortality (Figure 1) (5). Grade I meningiomas display a broad range of morphologic features and are considered histologically benign, with fewer than 4 mitoses/10 microscopic high-power fields (HPF). Nine subtypes of benign meningiomas are recognized by the WHO: meningothelial, fibroblastic, transitional (containing both meningothelial and fibroblastic components), psammomatous, angiomatous, microcystic, secretory, lymphoplasmacyte-rich, and metaplastic.

Grade II, also known as atypical, meningiomas are defined by the presence of 4–19 mitoses/10 HPF or 3 of 5 criteria: sheet-like growth, spontaneous necrosis, high nuclear to cytoplasmic ratio, prominent nucleoli, and increased cellularity. Meningiomas with two or less of the five atypical features are classified as grade I meningiomas with atypical features, and incur a higher risk of recurrence than benign meningiomas without atypical features (6). Two distinct histologic variants, clear cell and chordoid, are considered grade II meningiomas as well. In addition, the presence of brain invasion implies a similar recurrence rate and risk of mortality as atypical meningiomas (7).

Grade III meningioma is synonymous with anaplastic or malignant meningioma. Morphologically, they can resemble sarcoma or carcinoma, challenging pathologic diagnosis, and also include the papillary and rhabdoid histologic variants. Grade III meningiomas harbor a mitotic index of 20 or greater per 10 HPF, and classically lose markers of differentiation, such as epithelial membrane antigen. Patients with anaplastic meningiomas observe an aggressive clinical course of tumor recurrence and premature mortality.

## Challenges in Meningioma Management

The histopathologic classification of meningioma provides a powerful harbinger for its natural history. However, clinical outcome in a subset of patients belies the designated pathologic grade for the tumor. Improved understanding of the genomic underpinnings of meningioma offers new strategies for molecular stratification and rationally guided therapies. We first highlight some of the challenges facing meningioma management, then review recent advances in meningioma genomics, and draw upon lessons learned from other cancers.

### Limitations of Diagnostic Criteria

On initial detection of an extra-axial mass lesion consistent with meningioma on imaging, no reliable parameters exist to date for predicting tumor grade, and ultimately, biologic course. A number of radiographic metrics are under investigation, including nature of the tumor–brain interface, intratumor heterogeneity, lesion irregularity, intrinsic tumor diffusion and perfusion characteristics, and peritumoral edema, but all merit further validation. Furthermore, tumor location, such as parasagittal and falcine, may portend a more aggressive nature to the meningioma. Ultimately, mass effect leading to existing or impending symptoms, steadfast radiographic growth over a period of observation, and patient preference dictate the decision to intervene on a suspected meningioma.

Variations in operative philosophy, operative technique, and choice and timing of radiation permeate clinical practice. In general, maximal surgical resection without compromise of neurologic function imparts the most optimal prognosis for the patient. Standard of care typically invokes adjuvant radiation therapy for malignant meningiomas, with greater variability in the administration and timing of radiation for atypical meningiomas. This variability in management strategy for intermediate grade meningiomas is further complicated by shifts in diagnostic criteria over time (5, 8–10).

For example, application of the 2000 instead of the 1993 WHO guidelines results in a change in classification in up to 30% of high-grade meningiomas, often from a higher grade to lower grade (11). The 2007 WHO guidelines introduced less of a paradigm shift, but brain invasion remained ambiguous as a marker for atypical meningioma (12). The evolution of WHO grading scales associates with an improved correlation between grade and survival (13). However, inter-observer differences and representative sampling of select sections from large tumors may bias the final grading and, therefore, prediction of natural history. As with other CNS tumors, unbiased criteria for diagnostic arbitration, such as molecular signatures, can abet definitive stratification of tumor class. Furthermore, an association between such molecular signatures, tumor phenotype, and, ultimately, prognosis would improve initial planning for treatment interventions.

### Meningioma as an Invasive Tumor

Independent of tumor classification, the clinical course of meningiomas following surgical resection highlights its biologic proclivity for invasiveness. In Simpson’s classic analysis of symptomatic recurrence following resection of meningiomas, residual dural attachment and juxtaposition to venous sinuses – which serve as a potential haven for neoplastic cells in the absence of bulk tumor – were associated with significantly higher rates of recurrence (14). Furthermore, meningiomas with benign histopathologic features that invade the brain exhibit a similar likelihood of recurrence as higher grade, atypical, meningiomas. Thus, despite being the quintessential icon of CNS extra-axial tumors, the invasive potential of meningioma cells highlights an inherent limitation to debulking strategies and should be accounted for in therapeutic strategies.

### Intratumoral Heterogeneity

Surgical resection aside, radiation serves as a common adjuvant treatment for meningioma, especially in high-grade and recurrent tumors. A few biological agents, such as hydroxyurea and somatostatin inhibitors, have been trialed with limited success in meningiomas refractory to standard treatment modalities (15). These treatments rely upon the biologic response of non-senescent tumor cells. Additionally, the development of targeted pharmacologic inhibitors, as widely studied for systemic cancers and discussed below for meningioma, presumes a global distribution of the oncogenic driver or modulator target. The presence of intratumoral heterogeneity poses a fundamental impediment to the efficacy of these therapeutic strategies.

The observation of meningioma heterogeneity stems from a number of potential etiologies, including intratumoral necrosis, cystic degeneration, heterogeneous tumor cell expansion, imbalances in cell density, and hemorrhage. In particular, subclonal expansion within an admixture of functionally distinct cancer cells has been posited to account for incomplete treatment response, acquired and innate treatment resistance, and disease relapse for malignancies, such as glioblastoma and systemic cancers. Similarly, molecular and cellular heterogeneity is increasingly appreciated in meningioma (16), and may present a similar challenge to the development of therapeutic strategies.

These characteristics of meningiomas echo challenges posed by other tumors, some of which serve as exemplars in decrypting the molecular code toward a more unified front in diagnosis and treatment, as discussed below.

## Genomics of Meningioma

Meningioma represents one of the first tumors associated with a genomic driver, with the initial identification of *neurofibromin* (*NF2*), the causative gene for neurofibromatosis 2 (NF2), in which 50–75% of patients develop one or more meningiomas. Sporadic low- and high-grade meningiomas are also observed to harbor mutations, allelic inactivation, and loss of the *NF2* in ~40–60% of tumors, resulting in alteration of its protein derivative, Merlin (17–20). The development of meningiomas in *NF2*-knockout mice corroborates its role as an early oncogenic driver in meningioma tumorigenesis (21, 22).

**Figure 2:**
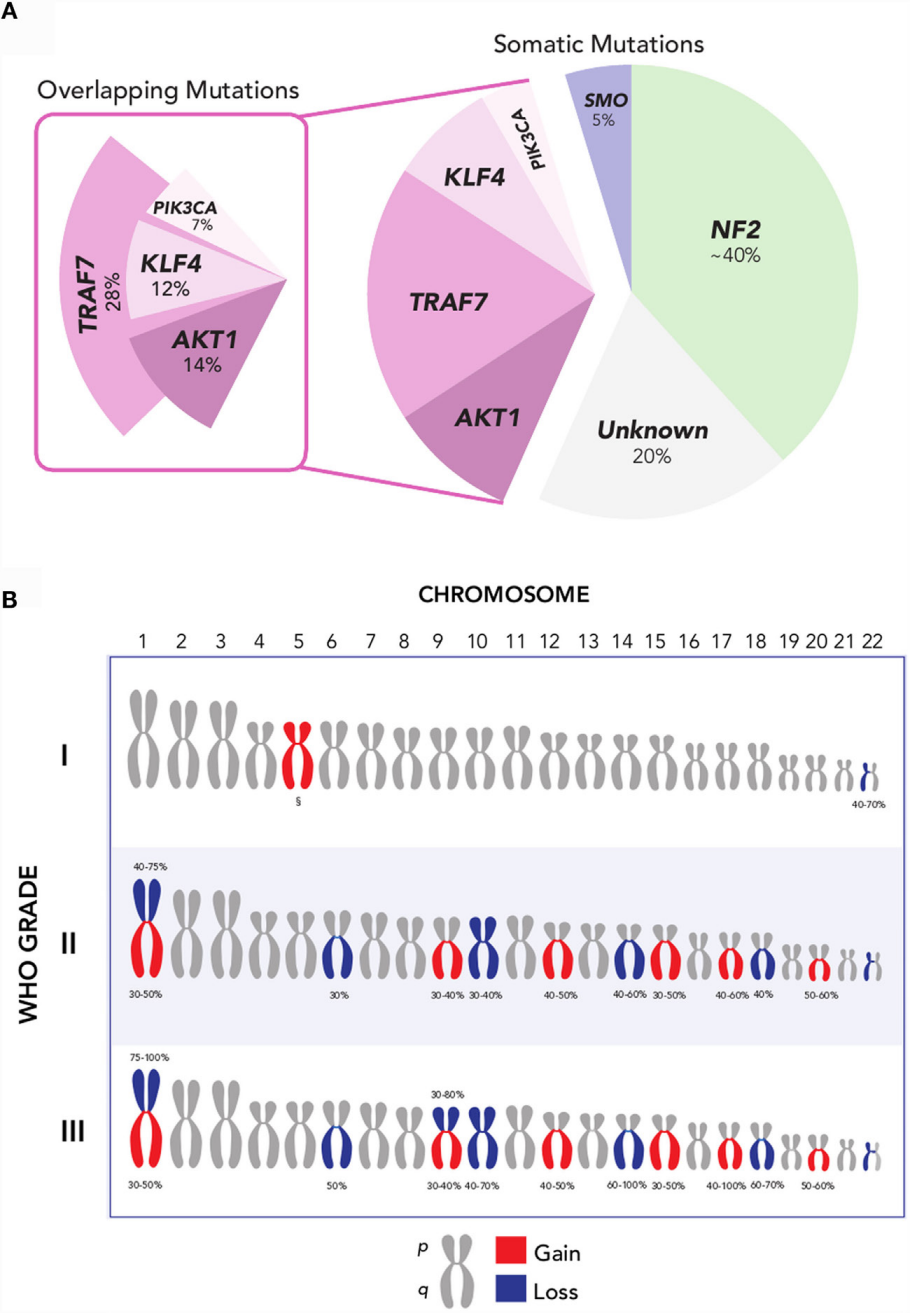
**(A)** Recurrent *NF2, AKT1, SMO, TRAF7, KLF4*, and *PIK3CA* mutations are collectively present in over 80% of grade I meningiomas. Mutations in *AKT1, KLF4*, and *PIK3CA* overlap with *TRAF7*, but not with each other, and largely occur in a mutually exclusive pattern with *NF2* and *SMO*. Oncogenic driver mutations remain unclear for ~20% of meningiomas [Data aggregated from Ref. (19, 20, 23, 41)]. **(B)** Recurrent chromosomal copy number alterations in meningioma. Chromosomal arm-level gains (red) and losses (blue) are observed with increasing frequency in higher-grade meningiomas, compared to grade I meningiomas. ^§^Polysomy 5 is observed in angiomatous subtype of grade I meningiomas [Data adapted from Ref. (5, 32)].

More recently, several additional recurrent somatic mutations have been identified through next-generation sequencing approaches, which are collectively present in ~40% of sporadic meningiomas (Figure 2A) (19, 20, 23). These genes are the pro-apoptotic E3 ubiquitin ligase *TNF receptor-associated factor 7* (*TRAF7*), the pluripotency transcription factor *Kruppel-like factor 4* (*KLF4*), the proto-oncogene *v-Akt murine thymoma viral oncogene homolog 1* (*AKT1*), the Hedgehog pathway *signaling member smoothened* (*SMO*), and the oncogene *PIK3CA*. Notably, mutations of these genes in meningiomas occur to large degree without concurrent alteration of *NF2* or loss of chromosome 22.

The most common of these is *TRAF7*, located on chromosome 16p13, which harbors a mutation in 12–25% of meningiomas (20). *TRAF7* mutations frequently co-occur with mutations in *KLF4, AKT1*, or *PIK3CA*, and are mutually exclusive with *SMO* and *NF2* mutations (20, 23, 24). A recurrent mutation in *KLF4^K409Q^*, located on chromosome 9q31 and resulting in a lysine to glutamine substitution at codon 409 (K409Q), represents the next most frequent somatic alteration observed to date – affecting ~15% of benign meningiomas. This may recapitulate embryologic mechanisms to spur tumor formation, given the role of *KLF4* as a transcription factor that promotes reprograming of differentiated somatic cells back to a pluripotent state in normal development (25). Another recurrent mutation in *AKT1*, located on chromosome 14q32, is observed in 6.8% of meningiomas and produces a glutamic acid to lysine substitution at codon 17 (E17K) (20, 26). *AKT1*^E17K^ mutation results in downstream activation of the *PI3K/AKT/mTOR* oncogenic pathway, rendering it targetable by selective AKT inhibitors, several of which are currently under investigation for the treatment of cancers of the breast, lung, and colon, among others (27). Oncogenic mutations in *PIK3CA* are observed in ~7% of non-*NF2*-mutant meningiomas, and occur mutually exclusive of *AKT1* and *SMO* mutations, although they frequently co-occur with TRAF7 mutations (23). Lastly, ~5.5% of benign meningiomas, or more than 10% of meningiomas without *NF2* alteration, express mutations in *SMO* (19, 20). These *SMO* alterations result in activation of Hedgehog signaling, another well-characterized pathway in cancer that is notably dysregulated in basal-cell carcinoma and medulloblastoma (28, 29). In basal-cell carcinoma, where over 90% of tumors have mutations in either *SMO* or *PTCH*, SMO inhibition has been particularly effective in the setting of locally advanced and metastatic disease (30). Inhibitors of SMO, AKT1, and PIK3CA hold promise as molecularly targeted pharmacotherapy in meningioma.

Collectively, these somatic mutations hold significant promise for advancing the molecular taxonomy of meningioma. However, ~20% of meningiomas remain without an identifiable oncogenic driver mutation to date (31). Beyond mutations, insertions, and deletions at the single nucleotide level, meningiomas harbor a classic constellation of chromosomal copy number alterations (Figure 2B). Monosomy 22 is the most common chromosomal change, observed in 40–70% of meningiomas, across all grades (7). Aside from loss of chromosome 22, the copy number landscape of benign meningiomas is typically neutral. One exception is the angiomatous subtype of grade I meningiomas, which notably express multiple polysomies across the genome, most commonly of chromosome 5 (32). In comparison, higher-grade meningiomas express a markedly higher burden of chromosomal losses and gain. These include frequent loss of chromosomes 1p, 6q, 10, 14q, and 18q, as well as gain of chromosomes 1q, 9q, 12q, 15q, 17q, and 20q (7, 33, 34). Among these, loss of chromosomes 1p and 14q is the most frequent cytogenetic abnormality observed in meningiomas after chromosome 22, affecting half of grade II and nearly all grade III meningiomas (33). Investigations into candidate oncogenes on these chromosomal arms have yet to elucidate clear drivers for meningioma tumorigenesis.

In addition to mutations and copy number alterations, epigenomic changes may provide another complementary biologic mechanism in meningioma development and progression (35). Overall, all existing evidence suggests a progression in genomic complexity in high-grade meningiomas.

## Applications of Molecular Taxonomy in Meningioma

These significant advances in our understanding of meningiomas provide an expanding toolbox to formulate a molecular taxonomy and explore novel therapeutic options for this surprisingly diverse tumor entity. This paradigm shift toward molecular taxonomy is inspired by examples from several tumor types, including glioblastoma, medulloblastoma, and ependymoma, where molecular stratification has transformed their diagnosis and management (36–38). Similarly, associations between molecular signatures with characteristic phenotypes, intracranial locations, tumor subclass, and clinical prognosis have begun to emerge as an increasing number of meningiomas are systematically characterized.

### Genetic Hallmarks of Meningioma Subtypes

The histologic subtype and location of meningioma associates with its molecular profile (Table S1 in Supplementary Material). Grade II and III meningiomas harbor an incremental complement of chromosomal alterations, as discussed above. Copy number gains, especially polysomy 5, are also characteristic of angiomatous meningiomas, a grade I subtype (32).

Focally, inactivation of *NF2*, through copy loss or mutation, occurs in 70–80% of fibroblastic and transitional meningiomas. By contrast, secretory meningiomas almost uniformly harbor mutations in both *TRAF7* and *KLF4^K409Q^* but not *NF2* (24), while meningothelial meningiomas are associated with *AKT1* mutations (26). Additionally, clear cell meningiomas are associated with loss-of-function mutations of *SMARCE1* in the hereditary multiple spinal meningioma syndrome and some cranial locations (39, 40).

Interestingly, genetic alterations also correlate with anatomic location in some meningiomas. Mutations in *SMO* or *AKT1/TRAF7* are most frequently observed in meningiomas of the anterior cranial base (19, 20). In comparison, convexity meningiomas are more likely to express *NF2* mutations and loss of heterozygosity of chromosome 22. The association between tumor location and genotype may aid candidate selection in future clinical trials that target specific oncogenic mutations.

### Predicting Clinical Outcome

Aside from the role of molecular biomarkers in abetting the diagnosis of meningioma, one fundamental question in the clinical management of meningioma patients is the risk of recurrence following surgical resection. There is particular ambiguity among grade II meningiomas, for which no consensus exists regards appropriate adjuvant treatment modality and timing. Recently, analysis of a cohort of atypical meningiomas following gross total resection revealed an association between increased chromosomal copy number alterations and risk of recurrence (41). By summing the incidence of broad copy number events across an aggregate pool of common chromosomal aberrations in meningiomas, this strategy bypasses the limitations of assessing isolated molecular candidates in meningioma oncogenesis and offers a rapid molecular appraisal of potential outcome through routine clinical cytogenetic testing. In other words, patients harboring grade II meningiomas with high chromosomal disruption, which may have a higher risk of recurrence, may benefit from closer surveillance or adjuvant therapies.

The validity of such molecular prognostication strategies remains to be proven in future studies. If corroborated, they may serve a powerful tool in counseling patients, guiding management decisions, and stratifying clinical trials.

### Designing Rational Strategies in Meningioma Treatment

Elucidation of critical oncogenic drivers in a number of cancers (e.g., *BRAF* in melanoma or *KIT* in gastrointestinal stromal tumors) has enabled targeted therapies in the so-called “mutation-to-drug” paradigm (42, 43). Such an approach is now feasible in meningioma with the recent identification of *AKT1, SMO*, and *PIK3CA* mutations, which opens the door for targeted pharmacotherapeutics in ~20% of grade I meningiomas. A clinical trial targeting AKT1 and SMO is currently underway for progressive meningiomas (NCT02523014).

While this genomically stratified trial augurs an exciting direction for refractory meningiomas that progress after standard therapy, other meningiomas that do not express these mutations, including most high-grade tumors, remain devoid of effective pharmacologic options. Furthermore, recognition of intratumoral cellular and molecular heterogeneity, which may foster resistant subclonal growth following targeted therapies, encourages investigation of alternative treatment strategies – such as immunotherapy (44).

Deployment of the innate and adaptive immune response offers an attractive option for genomically complex tumors, where presumably a higher neoantigen load is available for immune targeting (45, 46). Suppression of inhibitors of T-cell activation, known as immune checkpoints, has achieved durable clinical responses in several advanced systemic cancers (47). In grade II and III meningiomas that progress after surgery and standard radiation, a phase 2 clinical trial evaluating checkpoint blockade with nivolumab is anticipated to initiate (NCT02648997).

## Conclusion

Contemporary advances in molecular, genomic, epigenetic, and immune profiling has ushered a renaissance in the study of meningiomas. These systematic approaches suggest a molecular taxonomy that promises to influence diagnosis, disease classification, and, ultimately, clinical management. Furthermore, appreciation of shared biological characteristics between meningiomas and other CNS cancers – including invasiveness and intratumoral heterogeneity – may lead to an expansion of the therapeutic arsenal in the treatment of this increasingly disparate tumor.

## Author Contributions

WLB and IFD drafted the manuscript and supervised the study. MZ, WW, and YM contributed to data collection. All authors critically revised the manuscript and approved the final submission.

## Conflict of Interest Statement

All authors contributed to this article and attest to no conflicts of interest. All authors have no financial disclosures.
