# Supplementary material for: Meningioma Genomics: Diagnostic, Prognostic, and Therapeutic Applications
Source: Front Surg. 2016 Jul 6;3:40. doi: 10.3389/fsurg.2016.00040 (PMC4933705; doi:10.3389/fsurg.2016.00040)
Supplement: Supplementary file 1 [file table_1.pdf]

Supplementary Table 1. Genetic alterations associated with meningioma histologic subtypes.

Subtype	Associated Genetic Alteration	Inheritance Pattern
Meningothelial	*AKT* (E17K)	Sporadic
Fibroblastic, Transitional	*NF2*	Sporadic, Familial
Secretory	*KLF4* (K409Q), *TRAF7*	Sporadic
Angiomatous	Polysomy 5	Sporadic
Clear cell	*SMARCE1*	Familial
